# Cocaining Pulps for Removal

**Published:** 1897-12

**Authors:** 


ARTICLE V.

### COCAINING PULPS FOR REMOVAL.

The removal of dental pulps, which is never an undreaded operation by the patient, and not always an easy one for the operator, can often be accomplished very satisfactorily to both. Dr. J. X. Crawford, of Nashville, says: "Of all the operations in minor surgery demanding local anesthesia, the removal of a live pulp most demands it. Having decided through diagnosis by exclusion that it is clearly a case of pulpitis and that the pulp must be removed, put on the rubber dam, dry the cavity, wipe it out with bichloride of mercury 1-3,000, dry again, and repeat several times. When satisfied that it is sterilized, peel away the dentine carefully until you get a tiny exposure; then take a little muriate of cocaine, 10 per cent. solution, on a tiny wad of cotton, and lay it over the point of exposure. With your fingers form of wax a lid for the cavity of the tooth, fit it over and convert the tooth into a force-pump, forcing in the cocaine. Then trim away more dentine, enlarge the exposure, and work your pump again, forcing in more cocaine; you will find that you can remove the pulp painlessly and pull it clear out of the lingual canal, which will offer no resistance. Now before going any further, cork up the canal to prevent any debris from entering. Now work in more cocaine, and with a broach remove the pulp from the next canal and fill that up, and so on. That tooth is worth all the pains you can take with it, and you want to keep the canals in an aseptic condition, preventing the entrance of any debris from the dentine you will remove in preparing the cavity for filling. That pumping process will enable you to anesthetize the pulp so that you can remove it without pain to the patient, and you will not want any more arsenic. In case of hemorrhage, the application of the bichloride of mercury solution will arrest it at once."—*American Dental Weekly.*

